# Starvation resistance and tissue-specific gene expression of stress-related genes in a naturally inbred ant population

**DOI:** 10.1098/rsos.160062

**Published:** 2016-04-13

**Authors:** Nick Bos, Unni Pulliainen, Liselotte Sundström, Dalial Freitak

**Affiliations:** 1 Centre of Excellence in Biological Interactions, Department of Biosciences, University of Helsinki, Viikinaari 1, Biocenter 3, PO Box 65, Helsinki 00014, Finland; 2 University of Helsinki Tvärminne Zoological Station, Faculty of Biological and Environmental Sciences, J.A. Palménin tie 260, Hanko 10900, Finland; 3 Centre of Excellence in Biological Interactions, Department of Biological and Environmental Sciences, University of Jyväskylä, Survontie 9, Jyväskylä 40014, Finland

**Keywords:** gene expression, inbreeding, social insect, starvation, tissue specificity

## Abstract

Starvation is one of the most common and severe stressors in nature. Not only does it lead to death if not alleviated, it also forces the starved individual to allocate resources only to the most essential processes. This creates energetic trade-offs which can lead to many secondary challenges for the individual. These energetic trade-offs could be exacerbated in inbred individuals, which have been suggested to have a less efficient metabolism. Here, we studied the effect of inbreeding on starvation resistance in a natural population of *Formica exsecta* ants, with a focus on survival and tissue-specific expression of stress, metabolism and immunity-related genes. Starvation led to large tissue-specific changes in gene expression, but inbreeding had little effect on most of the genes studied. Our results illustrate the importance of studying stress responses in different tissues instead of entire organisms.

## 1. Introduction

In the wild, many species of animals face food shortage at least intermittently, making starvation resistance an essential fitness trait [1,2]. Prolonged food shortage enforces energetic trade-offs, where the starved individual has to allocate resources only to the most essential processes in order to survive. As a result, other energetically demanding functions, such as immune function, foraging activity and/or parental care can be impaired [3,4]. Indeed, starvation can suppress the expression of immunity-related genes [5], as well as many genes involved in insulin signalling [6] and endocrinology [7].

Studying the mechanisms behind organismal responses to external stressors and, in particular, to resource limitation helps us to understand how animals are adapting in a constantly changing environment. *Drosophila melanogaster* and *Caenorhabditis elegans* respond to starvation by entering a stress-resistant state, called adult reproductive diapause, in order to survive unfavourable conditions [8], which is characterized by arrested ovarian development, decreased metabolism, increased lipid deposition, resistance to stress and increased longevity [9]. Such physiological adaptation helps maintain organismal homeostasis under food deprivation, but can be reversed once more favourable conditions return. This enormous phenotypic plasticity in response to environmental and nutritional factors is probably subject to trade-offs between physiological pathways geared toward growth, reproduction and survival [8].

The effects of energy shortage could be exacerbated by other stresses, such as inbreeding. Interbreeding between related individuals occurs frequently in nature, despite the fact that various genetic and behavioural mechanisms have evolved to prevent this [10]. In many species, inbreeding reduces fitness [11–13], and one of the main reasons for this is the homozygosity of recessive deleterious mutations [14]. Evidence from studies on the fruit fly *D. melanogaster* suggests that inbreeding depression could present a form of genetic stress, which changes the regulation of genes and possibly decreases the efficiency for energy usage in inbred individuals ([15,16], but see [17]). Indeed, implications of inbreeding on metabolism have been found in, e.g. crickets [18] and the white-footed mouse [19].

In Hymenoptera, the effect of recessive deleterious mutations is thought to be less severe because these mutations will be purged when expressed in the haploid males [20]. However, social Hymenoptera, such as ants, are characterized by division of labour where different castes (i.e. workers, queens and males) conduct different tasks. Therefore, genes which are only expressed in females (i.e. queens and workers) would not be affected by purging in males [20,21]. Furthermore, an additional fitness cost of inbreeding can arise in the form of diploid males [22,23], as the gender of an individual ant is determined by a single sex-determining locus in many species ([24–26], but see [27]). Normally, diploid individuals turn into females, however, when individuals are homozygous at the sex-determining locus, they develop into (sterile) diploid males. As inbreeding reduces genetic variance, increased production of diploid males is expected, leading to reduced fitness. Furthermore, inbreeding could have an effect at two levels in social insects, namely that of the individual queen(s) and workers, and that at the level of the entire society [28].

However, inbreeding has been shown to carry crucial costs in terms of colony performance, such as colony longevity, sexual production and expression of immune responses. For example, previous work on a natural population of the ant *Formica exsecta* and two other *Formica* species found that on average, queens are less inbred than workers [29–31]. This has been hypothesized to be a result of either inbred queens having a reduced lifespan, as increased queen homozygosity reduces colony longevity or inbred brood being less likely to develop into queens [32]. In a later study on *F. exsecta*, both the total biomass of brood and the biomass of reproductive brood were shown to decrease when workers had higher levels of inbreeding [33]. However, although the biomass decreased, neither the total number of brood nor the total number of diploid brood was affected. This suggests that the shift was not due to differences in egg-laying rate or brood mortality, but probably due to reduced colony performance when workers are inbred, perhaps due to a lower metabolic efficiency [33], or inbred workers being less efficient at brood care.

Thus, as worker inbreeding levels are suggested to be of importance in relation to colony efficiency, possibly due to lower metabolic efficiency, we investigated whether inbreeding influences starvation resistance in ants. If inbred worker ants indeed have a lower metabolic efficiency, they could succumb to starvation earlier than non-inbred workers. However, the effects of starvation might not be equally visible in all tissues of an organism. Given that different organs fulfil different functions, measuring the changes in the gene expression in separate tissues instead of the whole organism provides more resolution when identifying the true effect of a stressor [34]. Therefore, we analysed the effect of inbreeding and starvation on tissue-specific gene expression, with a focus on stress-, metabolism- and immune-related genes. We compare gene expression patterns in the midgut to the rest of the abdomen. The midgut is of prime importance in this context as it is involved in absorbing nutrients and represents the first barrier against oral infections, while the rest of the abdomen is filled with fat body tissue, which is essential for energy storage as well as the systemic immune response [35].

## 2. Material and methods

### 2.1. Study organism

Nest workers from the interior of 23 colonies of *F. exsecta* were collected in spring 2012 on four islands outside of Tvärminne zoological station in the Hanko Peninsula, southwest Finland. These colonies are part of a genetically subdivided population, which has been surveyed since 1994 [30,32,33,36–38]. This population consists of *ca* 100 colonies alive each year, most of which are headed by a single reproductive queen (monogyne). Inbreeding occurs regularly, and as a result, colonies differ in their level of inbreeding. Colony-specific inbreeding coefficients (HL, homozygosity by locus, see [39]) have been estimated using 10 polymorphic microsatellite loci, weighed by the information content of the locus [32,33]. The 10 loci, on which the inbreeding estimates are based, all give highly consistent levels of inbreeding, and have been shown to be associated with several fitness traits [32,33]. The estimates have also recently been validated based on observed mating patterns in the same population and the same colonies [40]. Upon collection, the workers were brought to the laboratory and kept at room temperature (approx. 21°C) for the duration of the experiment. Workers were housed in plastic boxes (25 × 15 × 10 cm) lined with *Fluon*® (Whitford) in order to prevent the ants from escaping. Each box contained a thin layer of peat serving as extra nesting material. The ants were fed with Bhatkar–Whitcomb diet [41] and provided with water ad libitum. Owing to the fact that workers were collected from a natural population in the field, a possible effect of environment before collection exists. Collected workers were allowed to habituate for one week before the onset of the experiment.

### 2.2. Experimental design

#### 2.2.1. Experiment 1: effect of inbreeding on resistance to starvation

To investigate whether the level of inbreeding affects worker mortality upon starvation, 40 workers from each of the 23 colonies were, after the one week habituation period (see above), distributed equally between a control and an experimental group. Each group of 20 ants was housed in a small (7 cm Ø × 5 cm) box lined with *Fluon*® and containing a thin layer of peat (approx. 1 cm) in order to maintain humidity and serving as nesting material. The boxes were covered with a lid containing small holes. After being transferred to the experimental boxes, all the ants were fed for 1 day before the beginning of the experiment, in order to habituate to their new environment. After this, the control group was fed ad libitum with Bhatkar–Whitcomb diet [41], whereas the experimental treatment group did not receive any food. Both treatments were watered each morning if the nesting material was dry. Mortality was measured daily and dead ants were removed. The experiment lasted until every ant in both treatments had died (44 days).

#### 2.2.2. Experiment 2: tissue-specificity of gene expression upon starvation

After experiment 1, in order to study the effect of inbreeding and starvation on tissue-specific gene expression, we used 14 colonies out of the 23 we had collected. These colonies were chosen based on their HL value, to ensure as broad range of inbreeding (0.07–0.49) as possible. From each of these 14 colonies, 20 ants were distributed equally between an experimental (starvation) and control (fed) group, after which both groups were treated in the same way as experiment 1. On day 4, all individuals were dissected under a microscope (Leica Wild M3B, 10× magnification). Day 4 was chosen as the sampling point in order to maximize the effect of starvation, yet minimize mortality, as pilot data showed workers started dying quickly after 4 days of starvation. Dissection was done in a small dish containing a 1× phosphate buffered saline solution (PBS, Ambion), using two sets of sharp forceps in order to open the abdomen between the first two abdominal segments. The midgut was separated from the rest of the abdomen (henceforth called abdomen), and both were collected in separate 2 ml safe seal microtubes (Sarstedt) containing 200 µl TRISure (Bioline), a reagent for the isolation of RNA. In order to ensure enough material for RNA extraction, pooled samples of, respectively, five midguts or abdomens were used, resulting in two biological replicates per colony. Samples were frozen immediately and kept at −80°C until processed further.

### 2.3. RNA isolation and cDNA synthesis

Samples were allowed to thaw on ice, and total RNA was isolated according to manufacturer's protocol [42]. The RNA-concentration and quality of each sample was then determined using a NanoDrop 2000 Spectrophotometer (Thermo scientific). Before cDNA synthesis, any possible DNA contamination was removed using DNase treatment according to the manufacturer's protocol (DNase I, Thermoscientific). A total of 500 ng RNA was converted into single-stranded DNA using the iScript cDNA synthesis kit (BioRad) according to the manufacturer's protocol, after which the PCR products were stored at −20°C.

### 2.4. Focal genes, primer design and qPCR

Ten genes of interest were chosen based on their function, with a focus on immune-, stress- and metabolism-related genes (see electronic supplementary material, table S1). The immune-related genes chosen were defensin (Def), hymenoptaecin (Hyme), lysozyme C (LysC) and prophenoloxidase (PPO). These genes all code for effector molecules directly involved in immune response [43]. The stress- and metabolism-related genes chosen were heat shock protein 75 (HSP75), insulin receptor 1 (IR1), insulin receptor 3 (IR3) and VATPase (VATP) [35]. Furthermore, arylphorin (Aryl) and vitellogenin1 (VG, homologous to vitellogenin found in honeybees [44]) are known to have functions both in immune defence reactions and metabolism (nutrient storage and transport) [35]. Several candidates for housekeeping genes were tested with the subset of RNA samples for expression stability (elongation factor 1-alfa, ubiquitin and ribosomal protein 9). From these, only RPS9 showed stable expression, and was henceforth used as the endogenous control in qPCR. Real-time PCR oligonucleotide primers were designed using PrimerBLAST (http://www.ncbi.nlm.nih.gov/tools/primer-blast). Primers were designed by rules of highest maximum efficiency and sensitivity, and were based on *F. exsecta* transcriptome data from our Expressed Sequence Tag (EST) database [45].

Expression of the genes of interest was assessed using qPCR (CFX384 Thermal Cycler, BioRad). Each sample was run with two technical replicates, and the housekeeping gene RPS9 was present on each plate for every sample, to account for possible differences in sample handling and efficiency of cDNA synthesis. The samples were mixed with gene-specific primers and iQ SybrGreen supermix (BioRad), with following cycling conditions of 3 min 95°C, followed by 40 cycles of 15 s 95°C, 45 s 56°C and melt curve from 56 to 95°C with increment 0.5°C of 2 s step^−1^. Quality of the sample was based on the housekeeping gene RPS9, which was run for each sample.

### 2.5. Statistical analyses

All statistical analyses were done in R v. 3.0.2 [46]. Mortality during experiment 1 was analysed using the survreg function in the Survival package, including treatment as a fixed factor and inbreeding as a covariate, as well as colony as a random effect using the frailty command [47,48].

Delta Ct (ΔCt) values of each gene were transformed when necessary to avoid heteroscedasticity (for details, see electronic supplementary material, table S2). The effects of inbreeding, tissue (midgut versus abdomen) and treatment (starved versus control) on gene expression were tested separately for each gene using a linear mixed effects model (lme function from nlme package), using the ΔCt values of each gene as continuous variables, and a three-way interaction between the predictors allowed. Colony was added as a random effect to account for pseudo replication. We applied model selection according to Crawley [49]. *p*-Values (including those of dropped factors) were adjusted using the Benjamini–Hochberg false discovery rate (FDR) correction, in order to account for multiple testing.

## 3. Results

**Figure 1.**
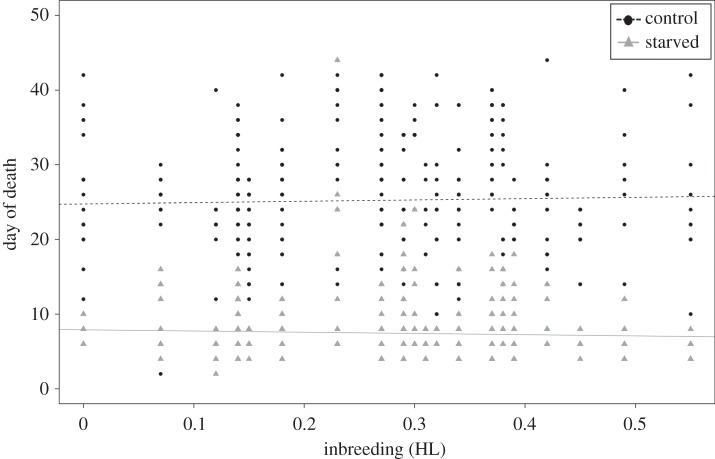
The effect of inbreeding on starvation-induced mortality. Starved ants died significantly faster than control ants, but no effect of inbreeding level was found.

As expected, starved ants died significantly earlier than fed ants (survreg, treatment: *z* = −23.29, *p* < 0.001, figure 1; electronic supplementary material, figure S1). Inbreeding did not have a significant effect on survival (survreg, HL: *z* = 0.01, *p* = 0.99), nor did it show a significant interaction with treatment (survreg, HL*treatment: *z* = −1.46, *p* = 0.14, figure 1).

**Figure 2.**
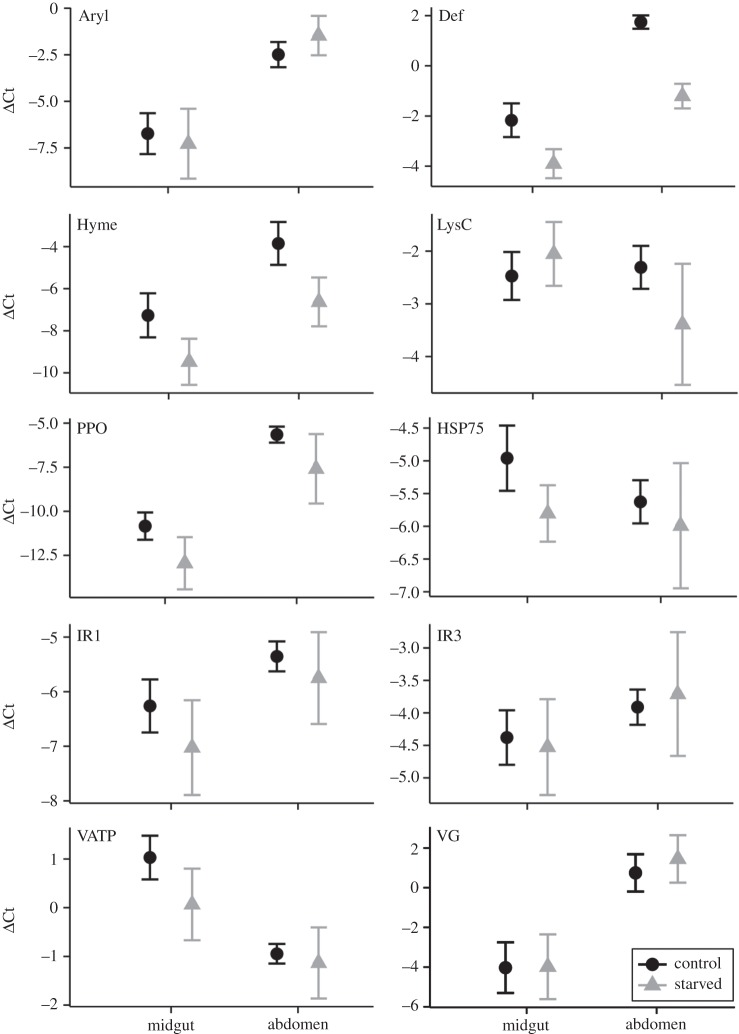
Effects of starvation-induced on tissue-specific gene expression. Expression of 10 different genes involved in immunity and/or metabolism. After correction for multiple comparisons, the effect of starvation was significant in Def, Hyme, PPO, HSP75 and VATP. The interaction between tissue and treatment was significant in Def. Statistical significances of each factor and interaction can be found in table 1. Data shown as mean ± 95% CI.

**Figure 3.**
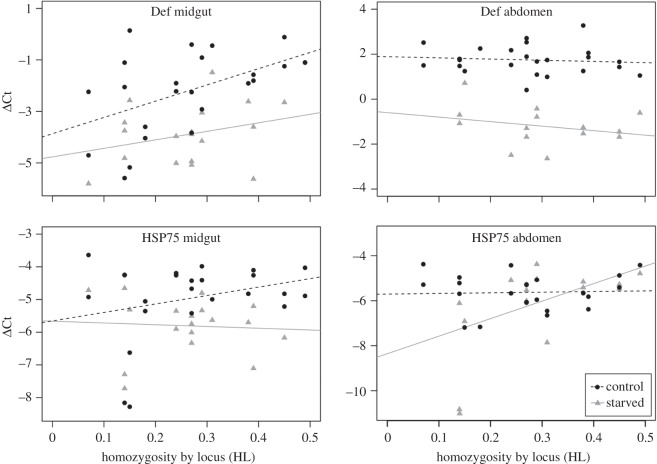
Effects of inbreeding on tissue-specific gene expression. Inbreeding had a significant interaction with tissue in Def. In HSP75, there was a significant three-way interaction between tissue, treatment and inbreeding.

The expression levels of the housekeeping gene were stable between treatments (lme{treatment}: *t* = 1.57, *p* = 0.12), but differed significantly between tissues (lme{tissue}: *t* = −3.30, *p* = 0.001), disallowing interpretation of the effect of tissue on gene-expression levels. This still allowed us to interpret the interaction between treatment and tissue (whether treatment has a different effect depending on tissue), as this value is based upon the direction of change in gene expression and not the baselines of expression. The effect of treatment was significant in five genes (lme{treatment}: Def, Hyme, PPO, HSP75, VATP, figure 2, table 1). None of the genes show a significant effect of inbreeding by itself, but Def showed a significant interaction between tissue and treatment, being more strongly downregulated in the abdomen upon starvation. (lme{tissue*treatment}, figure 2, table 1). Furthermore, Def was the only gene showing a significant interaction between tissue and inbreeding (lme{tissue*inbreeding}, figure 3, table 1). Thus, inbred ants have a significantly higher level of expression of Def in the midgut, whereas this effect of inbreeding is not apparent in the abdomen. None of the genes showed a significant interaction between treatment and inbreeding (table 1).

**Table 1.** FDR-corrected results of gene expression data. An overview of the gene expression results for (*a*) immunity-related genes and (*b*) stress- and metabolism-related genes. Significant *p*-values are bolded. Note that all *p*-values shown are obtained after FDR-correction. Also note that *p*-values regarding tissue cannot be properly interpreted due to the expression of the housekeeping gene differing significantly between tissues.

(*a*) Immunity	Aryl			Def			Hyme			LysC			PPO		
	d.f.	*F*	*p*-value	d.f.	*F*	*p*-value	d.f.	*F*	*p*-value	d.f.	*F*	*p*-value	d.f.	*F*	*p*-value
tissue	1,65	97.90	**<0**.**01**	1,62	256.18	**<0**.**01**	1,64	48.63	**<0**.**01**	1,63	1.65	0.42	1,64	159.34	**<0**.**01**
treatment	1,64	0.90	0.52	1,62	104.75	**<0**.**01**	1,64	29.42	**<0**.**01**	1,63	0.31	0.71	1,64	15.69	**<0**.**01**
HL	1,12	4.66	0.17	1,12	2.68	0.30	1,12	0.32	0.71	1,12	0.28	0.72	1,12	0.74	0.54
tissue : treatment	1,63	1.54	0.43	1,62	16.48	**<0**.**01**	1,62	0.76	0.54	1,63	5.34	0.09	1,62	0.78	0.54
tissue : HL	1,62	1.35	0.45	1,62	8.54	**0**.**02**	1,63	2.95	0.24	1,62	0.61	0.56	1,61	0.70	0.55
treatment : HL	1,61	1.34	0.44	1,61	1.08	0.49	1,61	0.15	0.82	1,61	0.13	0.83	1,63	3.88	0.17
tissue : treatment : HL	1,60	0.74	0.54	1,60	0.09	0.83	1,60	0.10	0.86	1,60	0.31	0.72	1,60	1.53	0.42

(*b*) stress and metabolism	HSP75			IR1			IR3			VATP			VG		
	d.f.	*F*	*p*-value	d.f.	*F*	*p*-value	d.f.	*F*	*p*-value	d.f.	*F*	*p*-value	d.f.	*F*	*p*-value
tissue	1,60	11.15	**0**.**01**	1,65	19.94	**<0**.**01**	1,65	7.92	**0**.**03**	1,63	84.26	**<0**.**01**	1,65	94.59	**0**.**00**
treatment	1,60	13.46	**<0**.**01**	1,64	3.55	0.20	1,64	2.86	0.24	1,63	7.69	**0**.**03**	1,64	0.02	0.91
HL	1,12	1.52	0.44	1,12	3.81	0.21	1,12	2.47	0.32	1,12	1.09	0.48	1,12	3.65	0.22
tissue : treatment	1,60	4.97	0.11	1,61	0.03	0.89	1,61	1.04	0.48	1,63	4.80	0.11	1,62	0.10	0.84
tissue : HL	1,60	1.07	0.48	1,62	2.73	0.25	1,63	2.06	0.34	1,62	0.62	0.56	1,61	0.00	0.99
treatment : HL	1,60	0.08	0.82	1,63	3.30	0.22	1,62	1.93	0.36	1,61	0.09	0.82	1,63	1.12	0.49
tissue : treatment : HL	1,60	6.59	**0**.**05**	1,60	1.31	0.44	1,60	0.84	0.53	1,60	1.61	0.42	1,60	0.05	0.86

Of the 10 genes studied, after correction for multiple testing, only the stress-related gene HSP75 shows a borderline significant three-way interaction between tissue, treatment and inbreeding (lme{tissue*treatment*inbreeding}, figure 3, table 1). In this case, inbreeding increases the level of expression upon starvation in the abdomen, but not in the midgut. This effect is different for non-starved ants, as their expression levels increase upon starvation in the midgut, but not in the abdomen. This suggests that depending on tissue, inbred ants react differently to the treatment compared with outbred ones.

## 4. Discussion

We show that in a natural population of the ant *F. exsecta*, inbreeding does not translate into higher mortality upon starvation, which is consistent with previous findings in *D. melanogaster* [17,50,51]. In agreement with this, inbreeding did not significantly affect the expression of 8 of the 10 genes studied. This stands in contrast with earlier results on other animals [15,16], in which the expression of many genes increased with the level of inbreeding, but is consistent with previous results in *F. exsecta* [52]. However, in a review by Armbruster & Reed [12], the authors found that 76% of the studies on inbreeding show an increase in inbreeding depression under stressful conditions. This leaves 24% in which inbreeding has no, or even has a positive effect under environmental stress [13]. For example, inbreeding increases relatedness, which may enhance social cohesion and altruism within groups [53], and inbreeding can drive beneficial genotypes to fixation [54]. Furthermore, the fact that we did not find negative effect of inbreeding in *F. exsecta* could reflect purging through haploid males. Indeed, previous studies on the same population also found no direct effects of inbreeding on worker immune systems, but did find indirect effects related to colony efficiency, such as lower biomass of brood and a lower number of new queens produced [38].

Irrespective of inbreeding, starvation itself influenced the expression of both immune-related and stress-related genes, mostly in the form of downregulation. This was particularly evident in the immune-related genes Def, Hyme and PPO. Def and Hyme are involved in the active degradation of bacterial cell walls [55,56], whereas PPO is involved in the melanization and encapsulation response. This suggests the presence of a physiological trade-off, as downregulation of these genes under stressful conditions could be a way of conserving energy when pathogens are not the acute stressor. This general downregulation upon starvation is in accordance with results found in *Drosophila* [57], where the response to starvation stress involves *ca* 25% of the genome, showing how strong a stress it is. By contrast, we found no change in the expression of IR1 and IR3. These genes are predicted to be essential in starvation regulation [58], and the lack of response may indicate the central role these genes play in metabolism. Furthermore, VG and Aryl also show no response to starvation, reinforcing the fact that they do not only have a role in immunity, but are also essential for organismal homeostasis, as downregulation of these genes might lead to the malfunction of crucial metabolic processes. The fact that many genes related to immunity are clearly downregulated upon starvation indicates that starved individuals could be especially vulnerable to stress caused by pathogen exposure. A link between starvation and immunity has previously been found in bumblebees, where workers' immune systems were artificially activated by the use of lipopolysaccharides and micro-latex beads, and led to a reduced survival upon starvation [3]. The stress-response related molecular chaperone HSP75 is particularly interesting as its expression increased in the abdominal tissue, but decreased in the midgut of inbred ants upon starvation (figure 3). This effect was borderline significant, however, and thus will require further verification. As sampling could be done only once per individual, we chose day 4 as a sampling point in order to maximize the effect of starvation while still minimizing mortality. However, it is important to note that because our gene expression data was collected on day 4, it is possible that other time points would show different patterns, as the chosen genes could potentially show acute phase changes.

To our knowledge, this is the first study to integrate the effect of inbreeding and starvation on tissue-specific gene expression in a wild population. We show, at the level of gene expression, that a possible trade-off between basic metabolism and immunity is more pronounced when resources are limited, an effect already visible 4 days after food deprivation. However, inbreeding had little effect on overall gene expression, but did affect 2 of 10 genes, one of which has importance in stress resistance (HSP75), while the other is involved in immunity (Def).

Our study shows hints regarding tissue-specific gene expression which would have been obscured if RNA was extracted from entire organisms, and would not correctly reflect the reaction of an animal to a certain treatment. An example of this is HSP75, where depending on the inbreeding level, ants showed contrasting responses in different tissues upon starvation. This suggests that inbreeding can indeed lead to tissue-specific responses leading to different trade-offs regarding resource allocation in stressful conditions.

## Supplementary Material

Figure S1: Survival of starved and control ants Food deprived ants die significantly faster than control ants.

## Supplementary Material

Table S1: Primer sequences used in qPCR Primer efficiencies calculated according to Biorad Real-Time PCR applications guide. Primers are considered efficient if their efficiency lies between 90% and 105%.

## Supplementary Material

Table S2: Transformations used for normalizing dCt values per gene. In order to avoid heteroscedasticity, transformation of the dCt values were done in nine out of ten genes.

## Data accessibility

Data and R-scripts used for analyses have been made available at dryad: http://dx.doi.org/10.5061/dryad.k3553.
