# Supplementary figures and images for: Starvation resistance and tissue-specific gene expression of stress-related genes in a naturally inbred ant population
Source: R Soc Open Sci. 2016 Apr 13;3(4):160062. doi: 10.1098/rsos.160062 (PMC4852642; doi:10.1098/rsos.160062)

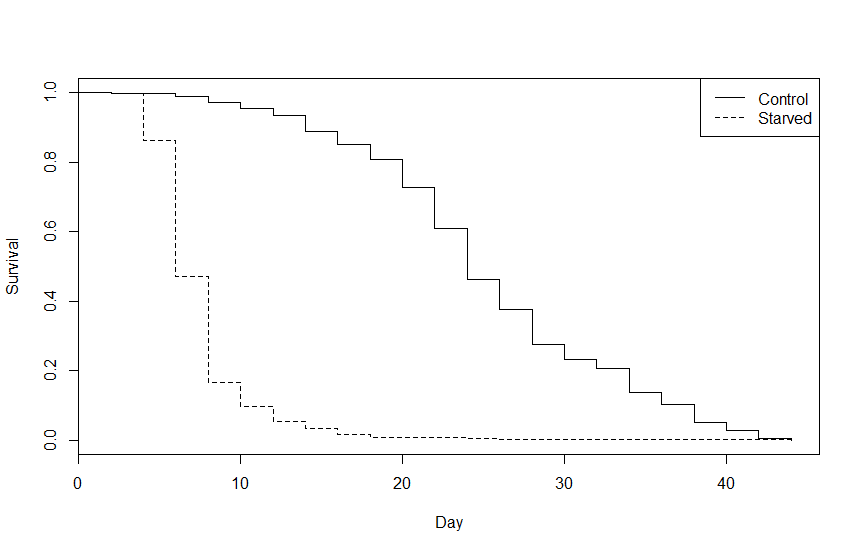

Supplement: Figure S1: Survival of starved and control ants Food deprived ants die significantly faster than control ants. [file rsos160062supp1.png]
