# Supplementary material for: Starvation resistance and tissue-specific gene expression of stress-related genes in a naturally inbred ant population
Source: R Soc Open Sci. 2016 Apr 13;3(4):160062. doi: 10.1098/rsos.160062 (PMC4852642; doi:10.1098/rsos.160062)
Supplement: Table S1: Primer sequences used in qPCR Primer efficiencies calculated according to Biorad Real-Time PCR applications guide. Primers are considered efficient if their efficiency lies between 90% and 105%. [file rsos160062supp2.docx]

**Table S1.** **Primer sequences used in qPCR**

Primer efficiencies calculated according to Biorad Real-Time PCR applications guide. Primers are considered efficient if their efficiency lies between 90 % and 105 %.

| **Gene** | **Forward primer** | **Reverse primer** | **Primer E%** |
| --- | --- | --- | --- |
| RPS9 | CCAACGGCATATTCGAGTAC | CAGTTTAATCCTCCTCTTCTTC | 98.53% |
| DEF | CGCACTTCCTAATGAAGATAAG | TTCCTCCCGTCTTTCCTCTGA | 93.80% |
| ARYL | ACT TAG AAC GCC TGT CCA AC | GAG ATT CCA TGT CCT CCA AG | 99.97% |
| HYME | TGG CAA AGA CGG TTT CAT CA | AAC CAC CAG TTA CAC CAA CC | 102.4% |
| LYSC | AGGTGGCATCTGTAACAAGC | CACCATCCTTCTTCTTCGTT | 91.63% |
| PPO | ACCTTCACAATCTCGGTCAT | TCCAACTGCTGTAGGCTGTA | 96.35% |
| HSP75 | AAGATAGGACTGCCAACGAA | GCAAGAGATGCAGAAGGTCT | 91.28% |
| IR1 | CGG TGT ACG GTG CTT TTC TA | GAA TCG GTC AAG AAC CCG AA | 91.28% |
| IR3 | GCG AGC GTT TTC TGT AGG TA | CCC AGG AGC TCG ATA ACA AC | 105% |
| VATP | GCCACAATGAGTTCACAAGA | CATGACAACAGGAATGATGG | 105% |
| VG1 | CGT TGC TTG TAG GGG ATA GG | AGG TTG TCA TCG TCA ACG AA | 98.33% |
