# Supplementary material for: Starvation resistance and tissue-specific gene expression of stress-related genes in a naturally inbred ant population
Source: R Soc Open Sci. 2016 Apr 13;3(4):160062. doi: 10.1098/rsos.160062 (PMC4852642; doi:10.1098/rsos.160062)
Supplement: Table S2: Transformations used for normalizing dCt values per gene. In order to avoid heteroscedasticity, transformation of the dCt values were done in nine out of ten genes. [file rsos160062supp3.docx]

**Table S2. Transformations used for normalizing dCt values per gene**In order to avoid heteroscedasticity, transformation of the dCt values were done in nine out of ten genes.

| **Gene** | **Transformation applied** |
| --- | --- |
| Aryl | Logarithm |
| Def | Square root |
| Hyme | No transformation |
| IR1 | Inverse |
| IR3 | Inverse |
| LYSC | Logarithm |
| PPO | Logarithm |
| VATP | Logarithm |
| VG2 | Logarithm |
| HSP75 | Inverse |
